# Modeling framework to demonstrate elimination of a vector population: Tsetse elimination in Chad

**DOI:** 10.1073/pnas.2524729123

**Published:** 2026-06-23

**Authors:** John Hargrove, Mahamat Hissene Mahamat, Moukhtar Aldjibert, Wilfrid Yoni, Djoukzoumka Signaboubo, Justin Darnas, Ernest Salou, Inaki Tirados, Albert Mugenyi, Priscille Barreaux, Philippe Solano, Antoine Marc Gaby Barreaux

**Affiliations:** ^a^ South African Centre for Epidemiological Modelling and Analysis, Centre for Epidemic Response and Innovation, School for Data Science and Computational Thinking, Stellenbosch University, Stellenbosch 7600, South Africa; ^b^Institut de Recherche en Élevage pour le Développement, Ndjamena 10001 BP 433, Chad; ^c^ Centre International de Recherche Développement sur l’Élevage en zone Sub-humide, Bobo-Dioulasso 01 BP 454, Burkina Faso; ^d^Programme National de Lutte contre la Trypanosomiase Humaine Africaine, Ndjamena 10001, Chad; ^e^ Unité de Formation et de Recherche en Sciences de la Vie et de la Terre, Université Nazi Boni de Bobo-Dioulasso, Bobo-Dioulasso 01 BP 1091, Burkina-Faso; ^f^ Liverpool School of Tropical Medicine, Liverpool L3 5QA, United Kingdom; ^g^ Ministry of Agriculture Animal Industry and Fisheries, Kampala 25601, Uganda; ^h^International Center for Insect Physiology and Ecology, Human Health Theme, Nairobi 00100, Kenya; ^i^ Interactions hôtes-vecteurs-parasites-environnement dans les maladies tropicales négligées dues aux trypanosomatidés, Univ Montpellier, Institut de Recherche pour le Développement, Centre de coopération Internationale en Recherche Agronomique pour le Développement, Montpellier 34398, France; ^j^ Centre de coopération Internationale en Recherche Agronomique pour le Développement, UMR Interactions hôtes-vecteurs-parasites-environnement dans les maladies tropicales négligées dues aux trypanosomatidés, Montpellier F-34398, France; ^k^International Center for Insect Physiology and Ecology, Animal health theme, Nairobi 00100, Kenya

**Keywords:** vector-borne disease, elimination, vector control, mathematical modeling, public health policy

## Abstract

Every year, over 700,000 people, particularly children under five, die from vector-borne diseases worldwide. Effectively controlling endemics and preventing new outbreaks requires an integrated approach that can lead to the elimination of both vectors and diseases. In the last two decades, integrating medical interventions and vector control has significantly reduced the incidence of Gambian Human African Trypanosomiasis (g-HAT), with the World Health Organization validating eight countries as having eliminated the disease as a public health problem. However, elimination of the tsetse vector has not been confirmed, leaving the possibility of re-emergence. We developed a six-step modeling framework to assess vector elimination by calculating: i) the probability of vector capture; ii) the probability of observing a series of zero catches, even without actual elimination; iii) the probability of natural elimination; iv) the probability of failing to detect a rebound; v) the reinvasion risk; and vi) the sensitivity analysis. Our case study is g-HAT in Mandoul, Chad, and the elimination of *Glossina fuscipes fuscipes.* We used vector control from 2014 to 2025 with no tsetse detected since 2018. We cannot yet conclude, with over 90% confidence, that tsetse has been eliminated from Mandoul, nor that any remnant population will be naturally eliminated. However, since vector control stopped in April 2025, we estimate that with continued sampling over the next 2 y, and no tsetse detected, elimination could be demonstrated with 99% confidence. Our multistep modeling framework can be applied to other vectors, providing policymakers with guidelines for ongoing and future efforts.

## Significance

The World Health Organization has set the elimination of transmission of several neglected tropical vector-borne diseases, including human African trypanosomiasis (sleeping sickness), as a target for 2030. We show that deliberate elimination of tsetse, the vector, is feasible and can be demonstrated. We draw on our large-scale intervention in Mandoul, Chad where 3000 insecticide-treated Tiny Targets were deployed between 2014 and 2025, with no tsetse detected since 2018. While small undetected remnant populations cannot be entirely excluded, they would rapidly rebound in the absence of control, becoming detectable. If no tsetse are caught over the next 2 y, it will confirm elimination. This illustrates a pathway for assessing and achieving vector elimination as a cornerstone of disease control and eradication.

Vector-borne diseases (VBDs) remain a major public health challenge worldwide with over 700,000 deaths annually, particularly among children under five (1). Sub-Saharan Africa bears the heaviest burden, where malaria, dengue, and other neglected tropical diseases (NTDs) affect millions of women and men. To successfully interrupt disease transmission, control approaches must address critical questions: when, where, how and with whom to act. When implemented effectively, integrated strategies can eliminate diseases to the point where the World Health Organization (WHO) validates them as Eliminated as a Public Health Problem (EPHP) (2).

Recent progress includes EPHP validation for gambiense Human African Trypanosomiasis (g-HAT or sleeping sickness), in Chad in 2024 (3), alongside seven other endemic countries including Guinea (4), and Côte d’Ivoire (5). After EPHP, the next step defined in the WHO roadmap on NTDs is elimination of g-HAT transmission by 2030, with the aim of achieving zero cases of the disease by that time (6). Integrated approaches must target not only diagnostics, vaccines, and therapeutics for the parasite and host, but also target arthropod vectors to reduce host–vector contact, and ultimately reduce and even eliminate transmission (5, 7–10).

Vector control successes include insecticide treated bednets and innovative housing approaches to control mosquitoes (7, 11) and Tiny Targets to control tsetse (*Glossina spp*), the sole vectors of HAT (12). In various settings and countries, these small insecticide impregnated pieces of fabric, Tiny Targets, have significantly reduced tsetse populations, lowering human–vector contact (5, 13, 14) and helping to halt g-HAT transmission (15). Vector ecology also strongly influences VBD epidemiology (16) and vector populations are highly sensitive to environmental and global change factors (17–21) which affect vector elimination prospects.

Despite progress, a critical question remains: how to know when elimination has been achieved and how to quantify the risk of error. While disease cases or vector captures may reach zero, confirming the complete elimination of either is a far more complex issue. The challenge lies in determining whether the absence of disease or vectors truly reflects elimination, or simply a limitation in our surveillance and detection capabilities. In some cases, the cessation of control efforts has led to a rebound in disease transmission. In some instances, stopping control has led to resurgence: halting control in the 1960s resulted in an estimated 300,000 cases of g-HAT in the 1990s, underscoring the need for a more robust understanding of vector population dynamics (2).

To address these challenges, we propose a mathematical and modeling framework, addressing the three core questions of VBD elimination: i) Can elimination, in theory, be achieved? ii) How will it be attempted in practice? iii) How do we know when it has been achieved? In our study, using tsetse and g-HAT as an example, we focus on the third question, considering diseases and vectors that can be eliminated with currently available methods (diagnostics, treatments, and tiny targets for vector control) and that for tsetse, elimination feasibility can be estimated from birth and death rates and the size of the starting population (22–24).

When a VBD elimination program reaches a stage where zero cases of disease are diagnosed, and/or zero vectors are captured, elimination of the disease and/or the vector may indeed have been achieved. Alternatively, this outcome may simply reflect the lower detection threshold of our sampling methods, below which parasites or vectors remain undetectable. If control efforts are halted while the disease and/or vectors persist—though undetectable with current tools—there is a risk of renewed transmission, rising case numbers, and ultimately a re-emergence of the vector, and/or the disease. We therefore investigate the theory underlying mathematical assessment of the probability that elimination has truly been achieved. We primarily focus on vector elimination, though clear parallels exist for disease elimination. Specifically, we explore mathematical approaches to estimate the risk incurred when declaring a vector population eliminated. This can be structured as a six-steps approach and modeling framework:

1. Before vector control is started, estimate the probability of capturing a vector with the surveillance tools to be used. This may be informed by existing literature when available, or by baseline mark-recapture, or other studies, to calculate the probability of capturing a vector. Seber (25) provides an excellent review of available methods and see Williams et al. (26) for more recent advances in the field.
2. Calculate the conditional probability of observing a series of zero catches given that at least one individual vector is still present. If that probability is sufficiently low, we conclude the vector has been eliminated (23);
3. If the vector population is very low but not yet eliminated, calculate the probability of natural elimination, purely by chance, without further control efforts (22, 24);
4. Use growth models to estimate the expected vector population at various times after the cessation of control efforts, assuming survival of at least one reproductive female. Failure to detect a rebound in the vector population after extended periods supports a conclusion that elimination has been achieved (23).
5. Calculate the probability of vector reinvasion to further support the demonstration of vector elimination (27).
6. Carry out a sensitivity analysis to estimate how our conclusions would be affected by variation in the assumptions regarding rates of vector development and mortality—both natural (encompassing environmental variations and climate change) and imposed (due to vector control).

As a case study of vector elimination modeling, we examine tsetse-transmitted g-HAT focusing on efforts to eliminate the tsetse species *Glossina fuscipes fuscipes* from the Mandoul focus in southern Chad (13)—a site of serious g-HAT outbreaks in the past. Both sexes of the tsetse fly are strictly hematophagous and are thus both capable of transmission. The disease is of particular interest because, while massive outbreaks occurred over the last two centuries, g-HAT seems now to be under control in many countries, with EPHP validated (3–5) and even elimination of transmission in some cases (15).

Tsetse provide a relatively simple, and tractable system for estimating the probability of vector elimination because of their slow, and predictable, reproductive biology. Each adult female produces only one larva every 8 to 10 d (28, 29). The larva deposited has approximately the same weight as its postpartum mother and contains the energy, microbiome, and materials required to pass through all of the final larval and pupal stages of metamorphosis, into an adult-sized teneral fly (28, 30, 31). Accordingly, following larviposition, the free-living pupa does not feed at all and reproduction can continue year-round, independent of the availability of environmental water (32). The production of a single offspring at each birth event, separated by roughly 10 d, means that the fate of individual offspring may be regarded as independent, which is an important assumption for probabilistic modeling. The low reproductive rate also means that population growth rates become negative if adult female mortality exceeds 4% per day. Sustained mortality at or above this level leads to elimination (22). In principle, tsetse populations should therefore be relatively easy to eliminate, particularly since they have never shown resistance to insecticide and their simple life cycle makes it feasible to calculate the probability that elimination can indeed be achieved.

Our research provides a modeling framework for assessing the likelihood of vector elimination, using *G. f. fuscipes* and g-HAT in Chad as a case study. This work contributes to ongoing elimination strategies by evaluating whether current evidence is sufficient to demonstrate that specific vectors, such as tsetse, have been successfully eliminated.

## Results

### Step 1: Probability of Capturing One *G. f. fuscipes* in a Biconical Trap in Mandoul.

**Fig. 1.**
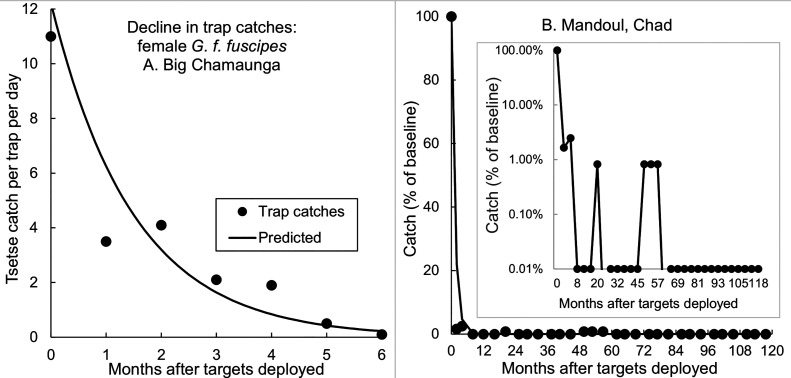
Decline in trap catches of female *G. f. fuscipes*: (*A*) Big Chamaunga Island, following the deployment of 30 Tiny Targets on the 1.5 km perimeter of the island. Fitted curve: Y = 12.22 e^−0.67^*^m^*. (*B*) Mandoul following the deployment of 2,713 Tiny Targets in the 840 km^2^ of the intervention area of the Mandoul focus (the actual area on which targets were deployed represents 45 km^2^, corresponding to the gallery surrounding the portion of the Mandoul River where tsetse were found). Fitted curve: Y = 100 e^−0.67^*^m^*. For both curves *m* is the number of months since the Tiny Targets were deployed. Details of calculations are available in Dataset S1. *Inset* Catches of *G. f. fuscipes* from traps deployed, between November 2013 and October 2023, in the Mandoul focus of southern Chad. Numbers are plotted as the log (base 10) of the catch; zero catches cannot thus be plotted.

Using data from Big Chamaunga Island, Lake Victoria, Kenya, we estimated the daily probability *P* that a female *G. f. fuscipes* in Mandoul is killed by a target or captured by an individual trap. To do this, we use the result that each 1% change in the daily mortality of adult female tsetse results, approximately, in a 10-fold change in the annual growth rate (33). On Chamaunga, catches of female *G. f. fuscipes* declined 10^4^-fold in 1 y after deployment of 30 Tiny Targets along 1.5 km of shoreline habitat (Fig. 1*A*) (14). This rate of decline is thus consistent with targets killing a proportion *p* = 0.04 (i.e., 4%) of adult females per day (33). Accordingly, the proportion *q* = 1-*p* that is not killed each day by any of the 30 targets is 0.96. The probability that a single target fails to kill a single fly in a day is then 0.96^(1/30) = 0.99864 and the proportion of the total population killed by one target is 1-0.99864 = 0.0014 or 0.14% per day. As a single biconical trap is estimated to catch about half as many tsetse as a Tiny Target (12), each trap should then capture (0.14/2) = 0.07% of the total fly population on the island per day.

We assume these kill and recapture rates when using Tiny Targets and biconical traps apply to our Mandoul situation since it involves the same tsetse subspecies, *G. f. fuscipes*. Implicit in this assumption is that targets cover all available habitat, such that each target’s 0.14% contribution is drawn from a local fraction of the total population. By the same logic, zero catches from a given trap reflect only the absence of tsetse in the immediate neighborhood of that particular trap.

In Mandoul, 145 *G. f. fuscipes* were captured in the 2013 baseline survey, and following the initial deployment of 2,713 Tiny Targets in March 2014, the follow up surveillance sessions—in April and June 2014—resulted in only 2 and 3 flies being captured, respectively (13)—suggesting that the population had already declined by c. 98%. The decline was so rapid that it is difficult to estimate the true rate of decline of the population (Fig. 1*B*). Nonetheless, the results are consistent with a population growth rate of −0.67 per month, equivalent to a yearly decline of exp (−0.67 × 12) = 0.000322 ≈ 10^−4^ per year, and the overall control effort killing c. 4% per day of the adult female population—as for the Big Chamaunga Island. Hence each target in Mandoul killed [1-(0.96)^(1/2,713)] = 0.000015 or 0.0015% flies per day and if the biconical traps used to sample *G. f. fuscipes* is about half as efficient as the Tiny Targets (12), each trap is expected to catch about 0.00075% per day of all flies in the Mandoul control area.

Since this level of mortality in Mandoul should guarantee eventual elimination, we expect that the continuous use of high densities of Tiny Targets tsetse should eventually lead to the elimination of the Mandoul *G. f. fuscipes* population. The following sections assess the probability that elimination has indeed been achieved—or, conversely, the risk that elimination has not been achieved, despite continued failure to detect any flies using the above calculated probability of capture per trap.

### Step 2: Probability of Zero Tsetse Catches, as a Function of the Numbers of Tsetse Surviving.

**Fig. 2.**
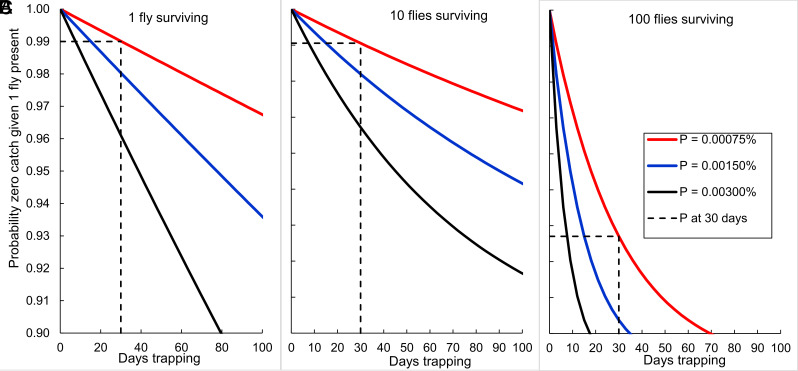
Probability of zero catch of tsetse, as functions of trap efficiency and numbers of days trapping—given that 1, 10, or 100 tsetse (*A*–*C* from left to right) have survived in the Mandoul area. The dotted lines show examples where 44 traps, each with 0.00075% efficacy, are run for 30 d. Notice that the range on the Y axis in *A* runs from 0.9 to 1.0, as opposed to 0.1 to 1.0 in *B* and *C*. Details of calculations in Dataset S2.

Suppose now that there was only a single tsetse remaining in Mandoul, and that the area is sampled using 44 biconical traps, every day for 30 d. The probability of observing zero catches from every trap on every day is then [(1-0.0000075)^44^]^30^ ≈ 0.99, so a 99% chance of having a false absence/failing to catch that surviving fly. Similar calculations show that even if the 44 traps were run for 160 d, there would still be a 95% chance of failing to catch the surviving fly. Moreover, even with a fourfold increase in trap efficiency, there would still be a 90% chance that 80 d of trapping would fail to catch a surviving fly (Fig. 2*A*).

If the number of tsetse surviving were 10, or 100, then the probability of observing a series of zero catches when some flies are still present obviously diminishes (Fig. 2 *B* and *C*). Nevertheless, if there were 10 surviving tsetse, 44 traps run for 30 d would still have a 91% chance of returning a series of zero catches/failing to catch any tsetse (Fig. 2*B*): and, with 100 surviving flies, there would still be a 37% chance of failing to capture a fly.

### Step 2’: Pursuing Vector Control and Vector Surveillance Until One Is 99% Confident of Tsetse Elimination by Relying on Probability of Capture in Surveillance Tools.

If one fly remains and vector control is continued and vector monitoring still relies on the same 44 biconical traps, then it would take 13,900 d to be 99% confident that one has not missed capturing that single surviving fly. Even with 4 times more efficient traps it would still take 3,475 d. If 10 flies remain it would still take 1,400 d with our current trap, and 348 d with a four times more efficient trap for a 99% confidence in tsetse elimination. Finally, if 100 flies remain, it would take 140 d with our current trap and 35 d with a four times more efficient trap to be 99% confident of tsetse elimination.

### Step 3: Probability of Natural Elimination of a Tsetse Remnant Population in Mandoul.

**Fig. 3.**
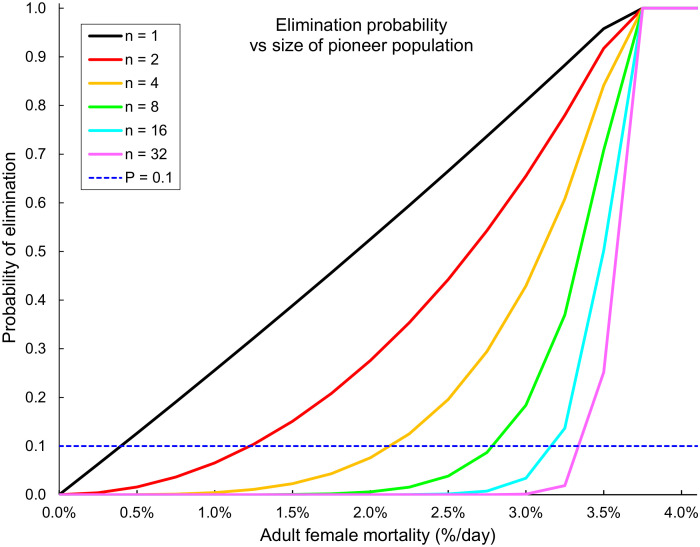
Probability that a small residual tsetse population is eliminated by chance, as a function of adult female mortality and the initial number of females in the residual population (different colored lines). Calculated from *SI Appendix*, Eq. **S1** (Dataset S8 with the following input parameters: Time to first ovulation, *u* = 7 d; Interlarval period, *I* = 9 d. Pupal duration, *T* = 30 d; Probability deposited pupa is female, α = 0.5; Probability female is inseminated, η = 1.0. Redrawn from ref. 22. Details of calculations in Dataset S3.

The previous sections underline the risk of inappropriately interpreting even a long series of zero trap catches as proof that the *G. f. fuscipes* population in Mandoul has been eliminated. If, however, we can at least be confident that the surviving population is small (10 or less) then what are the chances that such a population will be eliminated by chance? The results in Fig. 3 suggest that, in general, tsetse populations are remarkably resilient to being eliminated by chance.

For the Mandoul population, if indeed adult female mortality could be maintained at 4% per day, then any population would be eliminated. If, however, the imposed mortality fell to, say 2% per day then the probability that a remnant population of 10 inseminated female tsetse disappears by chance is <10%. And even a remnant of 16 such flies is only eliminated by chance with probability <10% if female adult mortality is about 3% per day. In general, therefore, we would be unwise to rely on a remnant population being eliminated by chance in Mandoul.

### Step 4: Allowing for the Detection of a Post-Vector-Control Rebound.

The expected growth rate of closed populations of tsetse, as a function of mortality and natality, are well established (22). The relationships are summarized in *SI Appendix*, Fig. S1, from which can be inferred the rebound of the hypothetical remaining tsetse population, following the removal of the Tiny Targets in the Mandoul area. At a low starting population number, implied by the inability to catch any tsetse with the surveillance system in place, we may safely assume that density-dependent mortalities among pupae and adults will be at a minimum. If we suppose that female mortality is at a conservative 2% per day, and that there are negligible losses among pupae and a 10-d pregnancy, then *SI Appendix*, Fig. S1 suggests that the population could increase by up to 100-fold in the first year. Such rates of increase have been approached for field populations of *Glossina pallidipes* in Kenya (34) and *Glossina morsitans morsitans* and *G. pallidipes* in Zimbabwe (35). We might then expect that the *G. f. fuscipes* population in Mandoul could grow to a minimum of 1,000 flies in 2 y. Even if the population increased by only 10-fold per year, the population should reach the 1,000 level in 3 y.

**Fig. 4.**
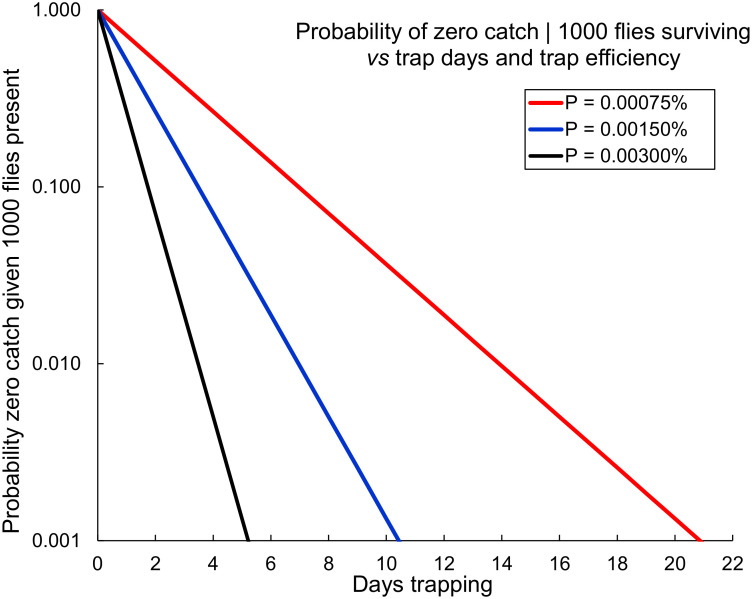
The probability of observing a series of zero catches of *G. f. fuscipes* in Mandoul after the removal of all Tiny Targets—given that the population has increased to a total of 1,000 flies before the sampling effort is initiated—plotted as a function of trap efficiency and numbers of days of trapping. Details of calculations in Dataset S4.

If the Mandoul population were to rebound to a level of 1,000 flies, 44 traps run for 3 wk (21 d) would catch at least 1 fly with 99.9% certainty (Fig. 4). That is to say, the probability of catching zero tsetse would be 0.001, or 0.1%. A zero catch over the whole 3-wk period would then imply there was negligible risk in concluding that elimination had been achieved. The above scenario—based on the assumption of sampling a population that was assumed to reach the level of 1,000 flies—might take 2 to 3 y to provide a decision that elimination has been achieved.

**Fig. 5.**
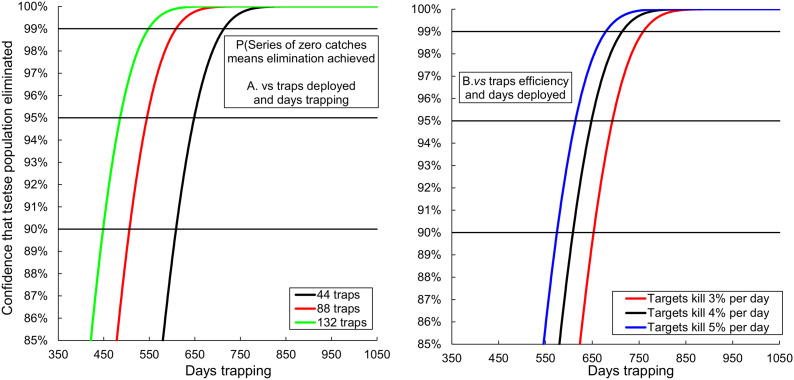
Estimates of confidence that series of zero trap catches support a conclusion that the Mandoul population of *G. f. fuscipes* has been eliminated. Calculated as a function of the number of days of sampling (trapping) and (*A*) The number of traps deployed, assuming that trap efficiency ε = 7.52 × 10^−6^ per day of being caught by any individual trap. (*B*) As a function of assumed trap efficiencies, per day, set according to assumption that Tiny Targets were killing either 3, 4, or 5% of adult females *G. f. fuscipes* per day in Mandoul, and that the efficiency of a trap is half of that of a Tiny Target. Details of calculations in Dataset S5.

The results in Fig. 5 suggest that a demonstration of elimination could be achieved in much less than 3 y. If the sampling procedure continues to involve the use of 44 traps, the initial probability of catching a fly will be small—and one would have <90% confidence that elimination had been achieved even after more than a year (550 d) of consecutive zero trap catches (Fig. 5). Thereafter, however, confidence levels grow very rapidly—reaching 90% and 95% by days 609 and 650, respectively, and 99% after 2 y.

Doubling the sampling effort, by using 88 traps every day, makes only a modest difference to the outcome; 1.5 to 2 y of zero catches would still be required to be 99% confident that elimination had been achieved. Increasing the trapping effort further would probably be counterproductive. If 132 traps were run daily, one could be 99% confident of elimination after 18 mo of zero catches (Fig. 5) but only if the traps were acting independently of each other. This assumption becomes increasingly unlikely as trap density increases (36).

### Step 5: Vector Elimination and Reinvasion Risk.

The closest population of tsetse, *G. f. fuscipes*, beyond the borders of Mandoul is around Timbéri, 50 km distant. Using the results of Hargrove and Lange (27) in modeling tsetse dispersal as diffusion in the plane, we estimate the probability that a tsetse fly could survive long enough to move between the two populations. The fly’s position (*x*, *y*, *t*) in time and space is defined by a normally distributed random variable. Mandoul and Timbéri are separated by a large distance (50 km), and the relative rate of diffusion is low (c. 0.04 km)^2^/d. Evaluation of *SI Appendix*, Eq. **S5** (*SI Appendix*, section 2) then shows that the probability that a fly can move, by active flight, between Timbéri and Mandoul in under 100 d is effectively 0, even if it survived for that period. For *t* > 100 d, the probability of completing the journey increases, but the probability that the fly survives this period decreases very rapidly. The danger of reinvasion due to diffusive movement is thus vanishingly low in any case.

We cannot, however, rule out the possibility that individual tsetse could be transported passively to Mandoul via, for example, cattle or motorized vehicles. The latter mode of passive reinvasion has been considered sufficiently important that tsetse control authorities in many African countries habitually searched for, and destroyed, tsetse in vehicles leaving tsetse infested areas. However, that is the case in national parks and conservancies for savannah flies which is vastly different from riverine flies and the Mandoul region in Chad. Hence, we regard as extremely unlikely the possibility of the passive reintroduction of numbers of flies sufficient to support a population resurgence in Mandoul. We are unaware of any documented example of such an event occurring anywhere in Africa. Moreover, there are no data available to support the estimation of the probability of such an event occurring. This uncertainty is not peculiar to the Mandoul situation—and is, in part, the reason that distinction is made between elimination, eradication, and extinction.

### Step 6: Sensitivity Analysis.

We investigated how our conclusions would be affected by variation in our assumptions regarding the rates of development in *G. f. fuscipes* and of mortality, either natural (environmental variations and assumptions on population stability) or due to the deployment of Tiny Targets. As regards the last-mentioned mortality, we are confident in using, for our study, the capture estimates obtained from Big Chamaunga Island, because they were obtained not only for any general riverine fly—but for the same tsetse subspecies, *G. f. fuscipes*, that occurs in Mandoul. It is difficult to justify our choice with other direct evidence because we can find no other study of the Palpalis group that provides any estimates of the probability of capturing a tsetse in a trap or killing one with a Tiny Target. In carrying out the following sensitivity analysis, however, we show that the observed decline in trap catches of *G. f. fuscipes* in Mandoul is consistent with the estimates of the efficiency of Tiny Targets and traps derived from the study on Big Chamaunga Island.

**Fig. 6.**
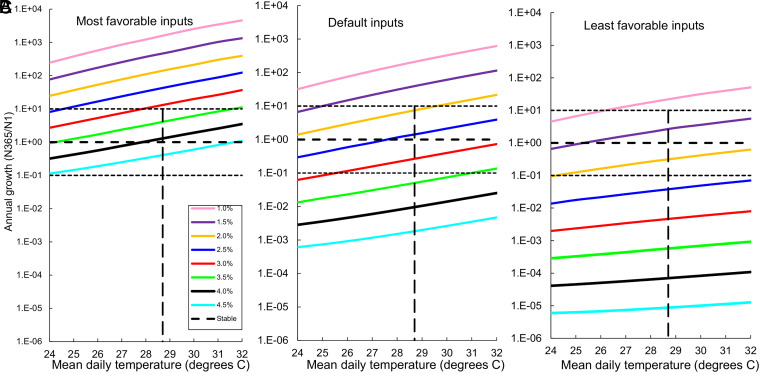
Growth of a tsetse population as a function of daily means of temperature and mature adult mortality (colored lines), with varying losses in preadult stages [three different scenarios: (*A*) Most favorable, (*B*) Default value and (*C*) Least favorable]. Assumed losses during premature adult stages were (*A*) 0%/pregnancy, 0%/day in pupae, and equal mortalities in young and mature flies; (*B*) 1%/pregnancy, 1%/day during pupal period, 3-times higher mortality in young than in mature adults. (*C*) 5%/pregnancy, 2%/day during pupal period, 5-times mortality in young than in mature adults. The three dotted lines mark population growths of 10, 1, and 0.1-fold per annum, respectively; the vertical dashed line is drawn at the approximate mean daily temperature for Mandoul of 28.7 °C. Details of calculations in Dataset S6.

Our sensitivity analysis is based on calculating, using *SI Appendix*, Eq. **S6** (Dataset S8), the population growth rate of a tsetse population, as a function of the parameters (Table 1) defining all relevant rates of birth, death, and development. We compared results when mortality levels—during pregnancy, pupal development, and ovarian category 0—were set at either 0% per day (most favorable), or at the default levels assumed for all of the calculations detailed above (our assumptions), or at the higher levels detailed in Fig. 6 (least favorable).

**Table 1.** Definitions of variables and parameters used for the calculations of growth rates and probabilities of elimination

Symbol	Definition	Units	Range of values assumed for *G. f. fuscipes* in Mandoul
*T_bar_*	Mean daily temperature (degrees C)	°C	24 °C to 32 °C
β	Fecundity, counting female larvae only	–	0.475 to 0.50
ρ	Probability deposited pupa female	–	α = 0.5
η	Probability adult female inseminated	–	η = 1.0 until near elimination
τ(*a*)	Pupal duration	days	20 to 29 d
µ(*a*)	Mortality rate during τ(*a*)	days^−1^	0 to 2% per day
ψ(*a*)	Daily survival probability during τ(*a*)	–	ψ(*a*) = e^−µ(^*^a)^*
τ(*b*)	Time emergence to first ovulation	days	7 to 8 d
µ(*b*)	Mortality rate during τ(*b*)	days^−1^	µ(*b*) = *g* µ(*c*) *g*≥1
ψ(*b*)	Daily survival probability during τ(*b*)	–	ψ(*b*) = e^−µ(^*^b)^*
τ(*c*)	Interlarval period (mature adults)	days	8 to 10 d
µ(*c*)	Mortality during τ(*c*)	days^−1^	Calculated from (*SI Appendix*, Eq. **S6**) if *r* preset
ψ(*c*)	Daily survival probability during τ*_c_*	–	ψ(*c*) = e^−µ(^*^c^*^)^
*r*	Population growth rate	days^−1^	Set at 0, or calculated from (*SI Appendix*, Eq. S6^*^)
ε	Trap efficiency (see text)		

If adult female tsetse experience a mortality of 2%/day then c.50% are expected to survive to the age of 35 d, which may be taken to approximate the mean life expectancy (37). By this time each female should have produced three larvae. If we allow a 33% loss during the pupal phase, the above scenario predicts an approximately stable population.

^*^For *SI Appendix*, Eq. **S6**.

We then used *SI Appendix*, Eq. **S6** to estimate the growth rate of the Mandoul *G. f. fuscipes* population—under the three different sets of assumptions shown in Fig. 6—for mortality rates, among mature adult females, varying between 1% and 4.5% per day. Our calculations provide, inter alia, estimates of what the adult mortality rate would have to be such that population had zero growth rate (*N*_365_ = *N*_1_)—which we assume was the case prior to the deployment of Tiny Targets. When default input parameters were assumed, the population had zero growth for mature adult mortalities, µ(*c*), of 2% per day at 24 °C, 3% per day at 32 °C and about 2.5%/day at the mean daily temperature of 28.7 °C (Fig. 6*B*). Under the most favorable circumstances for tsetse that we tested, the corresponding required mortalities increased to about 3.5% and 4.5% per day (Fig. 6*A*), whereas for the least favorable they were 1.5% and 2.0% per day at the lower and upper temperature bounds, respectively (Fig. 6*C*).

With reference to Fig. 6*B*, where we use the default input parameters, a stable population is consistent with a 2 to 3% natural mortality, µ(*c*), among mature adult females. We thus expect a total mortality of roughly 6 to 7% when the targets are deployed. We now ask what these different scenarios would imply about the proportion of the decline in fly catches that should be attributed to the Tiny Targets and, accordingly, what proportion of the population was being killed each day by these targets. It has been argued that each 1% per day of added mortality among mature adult female tsetse reduces the annual growth rate by a factor of approximately 10 (33). The results in Fig. 1*B* are, accordingly, consistent with adult female *G. f. fuscipes* in Mandoul experiencing an additional mortality of *c*. 4% per day due to the Tiny Targets. Notice that this approximation holds for all three scenarios in Fig. 6—as long as the population had zero growth rate prior to target deployment. Thus, while different levels of natural mortality among mature adult female would be necessary in each of the three scenarios, to ensure a zero growth-rate in the uncontrolled population, it would always be necessary for the targets to kill an additional ~4% per day to replicate the results in Fig. 1*B*.

The treatment above applies only to a population with zero growth rate. It is, however, possible that climate change over the past decades might have resulted in a natural decline in the Mandoul tsetse population—which might have escaped attention, given that the population of *G. f. fuscipes* was already so low prior to the deployment of Tiny Targets. Consider a population living at 28.7 °C—and declining in number at a rate of 10-fold per year—i.e., *N*_365_ = 0.1*N*_1_. Using the default input parameters this would imply that the adult female mortality rate was 3 to 3.5% per day (Fig. 6*B*). For Tiny Targets to reduce the population at the observed rate, it would only be necessary for them to kill 3% of the adult females per day to replicate the results seen in Fig. 1*B*. This would not affect the theoretical probability of elimination, since the same proportions of flies are still being killed overall. Our estimate of the proportion killed per day by the targets—as opposed to natural causes—would, however, be reduced. Likewise, our estimate of the proportion of the population captured per day by a trap would also be reduced and this would mean an increase in the number of trap/days with zero catches required to achieve any given level of confidence that elimination had been achieved.

It seems unlikely that the Mandoul tsetse population was expanding prior to target deployment. Such a situation, had it been carrying on for any length of time, should undoubtedly have been noticed by the local population—due to an increase in fly biting rate and/or cases of trypanosomiasis. Nonetheless, if we suppose that the population had been increasing by a factor of 10 per year, then—using the same logic as applied above—we should expect that, if the default input parameters apply, the adult mortality prior to target deployment would have been reduced to slightly more than 1.5% per day. To replicate the results in Fig. 1*B* it would then be necessary for the Tiny Targets to kill 1.5 + 3.5 = 5% per day (Fig. 6*B*), i.e., a greater proportion than under the assumption of the default parameters. By extension, the traps would be catching a larger proportion than we assumed, and it should be possible to conclude earlier, following a series of zero trap catches, that elimination had been achieved.

Given the foregoing analysis, we investigated the effect of allowing the assumed kill rate of adult female *G. f. fuscipes* to take values of 3% and 5% per day, rather than the default value of 4%. This meant that there was, overall, a 100-fold change in the annual decline in population numbers, and that the implied probabilities of capture in a trap were 5.61 × 10^−6^, 7.42 × 10^−6^ and 9.45 × 10^−6^ for target kill rates of 3, 4, and 5%, respectively. The predicted increase in the probability of being able to declare that elimination had been achieved varied little with the above changes in assumed kill rates and trap efficiency ε (Fig. 5*B*). Indeed, we reach 99% of confidence that tsetse are eliminated if no rebound is detected after 759 d, 725 d (about 2 y, our default above), or 690 d of zero catches when we estimate population were decreasing, stable, or growing before the start of vector control and with target kill rates of 3, 4, and 5%, respectively.

### Density-Dependent Effects.

Within 12 mo of the deployment of Tiny Targets in the Mandoul area, catches of *G. f. fuscipes* declined to essentially undetectable levels (Fig. 1*B*), at which point density-dependent mortality would be expected to decline to minimal levels in all life stages. Identifying the life stage(s) at which density dependence is the most important has proven difficult in field settings. To date, convincing empirical evidence exists only for density-dependent mortality in *G. f. fuscipes* pupae (38). Regardless of its mechanistic basis, however, density-dependent mortality cannot fall below zero, and thus cannot offset sufficiently high imposed mortality. Consequently, sustained control at adequate intensity will lead to elimination irrespective of density-dependent effects (33). Indeed, when population density is extremely low the tsetse population will be at increased risk of elimination due to the Allee effect (39, 40), which will act via reductions in the parameter η—the probability that a female fly becomes inseminated (Table 1).

## Discussion

No *G. f. fuscipes* have been captured in Mandoul since 2018, following a vector control program that began in 2014 and ended in 2025. It is not yet possible to conclude, with more than 90% certainty, that the population has been eliminated, owing to the low probability of catching *G. f. fuscipes* with current trapping methods. Nonetheless, satellite images and community reports (source: Mahamat M.H., Aldjibert M., and Yoni W., 2025, pers. comm.) strongly suggest that tsetse no longer pose a threat: crops are now cultivated in areas previously infested with tsetse and new human settlements are emerging along the Mandoul river. These developments further support the decision by the Chad tsetse control team to remove all Tiny Targets in early 2025. If current levels of surveillance continue for two more years without detecting a single *G. f. fuscipes*—then our results suggest that the authorities will be able to declare elimination with 99% confidence. This would represent one of the few modern examples, worldwide, of successful vector elimination. Based on our sensitivity analysis we are confident regarding the robustness of our results. Variation in the assumptions relating to rates of vector development and mortality—both natural (encompassing environmental variations and climate change) and imposed (due to vector control)—do not strongly impact our predictions and conclusions.

If a small number of tsetse persist, their population should increase once the mortality imposed by the Tiny Targets is lifted, making detection more likely. This represents a risk-reward situation: while risks appear low given over 6 y without captures, Tiny Targets could be redeployed to quickly suppress any remnant population. The rewards, by contrast, are substantial—cost savings from halting control and the prospect of eventually ending all monitoring. Without stopping control, it would take an impractical (and expensive) 38 y of continuous trapping with current methods to be 99% confident of elimination, and even longer with the present regime of a few trapping days per year. The near-zero risk of reinvasion from neighboring tsetse populations, combined with the improbability of accidental introduction via livestock or motorized transport, further supports cessation of control. No other tsetse population or species appear to be able to recolonize the area vacated by *G. f. fuscipes.*

Our study highlights the difficulty of demonstrating complete elimination of an isolated vector population when monitoring tools cannot reliably detect individuals at vanishingly low population density. Moreover, we show that the problem still exists even if we are less conservative in allowing the detection rate to increase by a factor of 4. The problem is exacerbated for Palpalis group tsetse, including *G. f. fuscipes*, because they are less strongly attracted by host odors, to traps and targets, than Morsitans group flies (41). Moreover, Rayaisse et al. (42) who carried out the most comprehensive study on the subject, found that only ~50 % of the flies attracted to the vicinity of a trap are caught by it. Nonetheless, trap catches could be doubled using synthetic host odors. Unfortunately, such data do not exist for *G f. fuscipes* captures; moreover, the improved catch of Morsitans flies with electric traps (43, 44) was not observed in palpalis flies (42). Recent innovations are devices such as the red trap (45), with increased efficiency for biting flies such as *Stomoxys*, and biodegradable traps, such as the Bioflytrap (46). No innovations in the last decades have, however, improved the capture rates for Palpalis group flies and none are in the pipeline, to the best of our knowledge. Hence there are few alternatives to the tools used in this study. Together, these observations underscore that the principal constraint on elimination validation in such settings is not analytical uncertainty, but the fundamental performance limits of available surveillance technologies.

The problem of demonstrating elimination also scales with area size, as shown in the large-scale efforts to eliminate *G. m. centralis* in Botswana. There, contiguous blocks of 16,000 sq km in the Okavango Delta were sprayed in 2001 and 2002, and no tsetse have been caught since the completion of the second year of spraying (47). Trap and fly-round sampling methods would not, however, have detected tsetse at population densities of 1 per sq km, nor could such sampling methods cover the full habitat. The conclusion of elimination relied on the expectation that any surviving tsetse would have rebounded to detectable levels within 2 y.

Historically, such a “wait and see” approach relying on years without tsetse captures or reported cases of disease has underpinned declarations of elimination, with the passage of time without new cases providing sufficient evidence of local eradication. This is the basis for accepted cases such as *G*. *p*. *palpalis* on Principe (48), *G. pallidipes* in Zululand (49) and *G. m. morsitans* in Umfurudzi Game Area of Zimbabwe (32) where elimination was inferred without long-term systematic sampling. The passage of years, and then decades, in which no tsetse and no cases of trypanosomiasis were reported, simply made it clear that the flies had indeed been eliminated. In contrast, very small or isolated areas allow quicker demonstration of elimination, as shown for *G. pallidipes* and *G. m. morsitans* on Antelope Island, Zimbabwe (35), and *G*. *austeni* on Unguja Island (50). Mathematical approaches, including landscape genetics (51) or species distribution modeling (52) have also been developed to help identify such target areas for disease or vector elimination.

Regardless of the scale or surveillance strategies, our framework provides a formal, probabilistic method to demonstrate vector elimination. This creates direct linkages to existing international processes: WHO’s elimination as public health problem (EPHP) (2) and elimination of transmission (EoT) for g-HAT, FAO’s Progressive Control Pathway for Animal African Trypanosomiasis (AAT) (53), and WOAH’s recognition of AAT-free status. All are aiming for a greater integration of mathematical frameworks to move beyond reliance on the “wait and see” paradigm. Discussions are underway on incorporating our approach into official WHO and FAO procedures.

Although developed for tsetse, the framework is general and applicable to any vectors or even populations of beneficial species, be it plants or animals, at risk of elimination, or even extinction. Our results are therefore relevant to public health experts, policy makers, and conservation practitioners.

In estimating the probability that the Mandoul population of *G. f. fuscipes* has been eliminated, we did not account for the possibility that long-term use of Tiny Targets may have induced behavioral change in tsetse, such as avoidance of these attractive systems. Similarly, selection pressure could have resulted in surviving flies feeding preferentially on host animals that can be accessed with reduced movement, thereby lowering the likelihood that flies are killed by Tiny Targets. There is, however, no evidence that suggests such behavioral or ecological shifts have occurred, and any potential impact on our results would be minimal. Importantly, the selection pressure imposed by our 44 surveillance traps is far weaker than that imposed by 2,713 Tiny Targets, meaning that monitoring is less likely to be affected by the evolution of resistance than the vector control efforts.

We also adopted a conservative approach in our calculations to minimize the risk of incorrectly demonstrating elimination in Mandoul and to ensure our conclusions are not overly optimistic. For instance, in calculating the probability that a small surviving population of tsetse would not be eliminated by chance, we disregarded the possibility that adult virgin females might die before successfully mating linked to the Allee effect (39, 40), a factor that would further reduce the chance of persistence. In this respect, our estimates represent a worst-case scenario of the results obtained by the tsetse control team in Mandoul.

We were also conservative when considering a scenario and parameter values that were neither the most nor the least favorable to tsetse, demonstrating the robustness of our results. Our sensitivity analysis allowed substantial variation in a large number of parameters associated with tsetse population growth and control and encompassing environmental variation and climate change, without seriously affecting the conclusions of our modeling. These results strengthen our argument that the risks of removing the Tiny Targets are outweighed by the benefits of that policy.

## Conclusion

Whether or not tsetse have already been eliminated from the Mandoul area, Tiny Targets have successfully reduced tsetse population densities by several orders of magnitude to undetectable levels. In 2024, WHO validated elimination of g-HAT as a public health problem in Chad, marking the operation a success (3). With the removal of Tiny Targets, continued monitoring will provide a definitive answer regarding the elimination of tsetse in the Mandoul region. More broadly, the multistep theoretical approach developed in this study offers a rigorous and robust method for demonstrating vector elimination even under varying environmental conditions or climate change and the linked uncertainty on vector population stability, mortality, and growth rates, and the efficacy of vector surveillance and control. This framework is applicable to other disease vectors and provides guidance for policymakers and health authorities in planning, evaluating and sustaining future elimination efforts.

## Materials and Methods

### Modeling Framework for Assessing Vector Elimination.

**Fig. 7.**
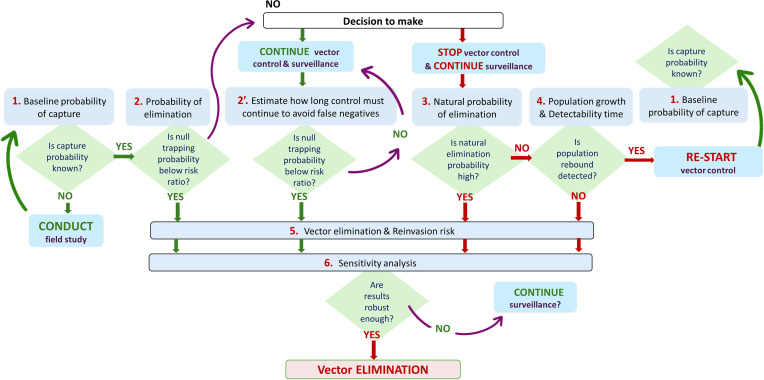
Modeling framework and decision tree for declaring vector elimination: Step 1. Baseline work to assess the probability of vector capture (probability from experiments or estimated from the literature); Step 2. Calculate the conditional probability of observing a series of zero catches given that there is still at least one individual vector present. If that probability is sufficiently low, we conclude the vector has been eliminated (one only enters step 2 once zero catches of vectors start to be observed in the surveillance system); alternatively step 2’, calculate how long vector control, and surveillance, should continue in order that the risk of obtaining a false negative is acceptably small; Step 3. If the vector population is very low, even if not yet eliminated, calculate the probability of the remnant vector population being eliminated naturally, purely by chance, without further control efforts; Step 4. Use growth models to estimate the expected vector population at various times after the cessation of control efforts, assuming the survival of at least one reproductive female. Failure to detect a rebound in the vector population after protracted periods supports a conclusion that the vector has already been eliminated. If no rebound is detected then the control team must decide whether vector control should resume or whether other disease control actions are necessary (depends on hosts cases surveillance, disease control status, and logistical and budgetary constraints); Step 5. Vector elimination can then be declared with a specified risk, and the risk of tsetse reinvasion can also be evaluated; Step 6. Sensitivity analysis to estimate how variations in the assumed rates of development and mortality—both natural (encompassing environmental variations and climate change) and imposed (due to vector control)—in the vector population could affect our conclusions. Details on the equations behind these steps are in *SI Appendix*, section 2.

Using a decision tree (Fig. 7), published methodology (22–24), and our modeling framework, we use up to six steps, in sequence, to estimate the probability that a vector population has been eliminated in a specific area, and the best course of action to progress to elimination. The following points provide a synopsis of the six-step procedure. Details are provided for Mandoul in *SI Appendix*.

1) Baseline probability of capture with a surveillance tool: if there is no relevant existing literature conduct mark-recapture, or other, studies to calculate the probability of capturing a vector. Alternatively, calculate the theoretical probability that a given control method could eliminate a vector population that is isolated (i.e., closed to all in- and out-migration) (32) and infer the probability of capture with the surveillance tool from the vector control tool efficacy.
2)  Probabilistic modeling of elimination: apply a probability model to the results of trapping efforts to reject the null hypothesis that vectors are still present in the area.
3) Natural probability of elimination: estimate the probability that a very small residual population will be eliminated by chance.
4)  Probability of detecting a rebound: use a model of tsetse population growth to estimate the time required for a very small remnant population to become detectable by trapping.
5) Vector elimination and reinvasion risk: evaluate whether the vector population has been successfully eliminated with a specified level of risk, while also accounting for the potential threat of reinvasion.
6) Sensitivity analysis of the framework: use parameter ranges appropriate for the vector species and ecology/location under consideration. The parameters relate to population growth rates (i.e., vector birth and mortality rates) and the probability of capture or being killed by vector surveillance or control tools. Allowing the parameters to vary makes the prediction more robust to any reporting or experimental error, environmental variation, and climate change.

### Applying the Vector Elimination Modeling Framework to Our Case Study in Mandoul, Chad.

#### Study area.

**Fig. 8.**
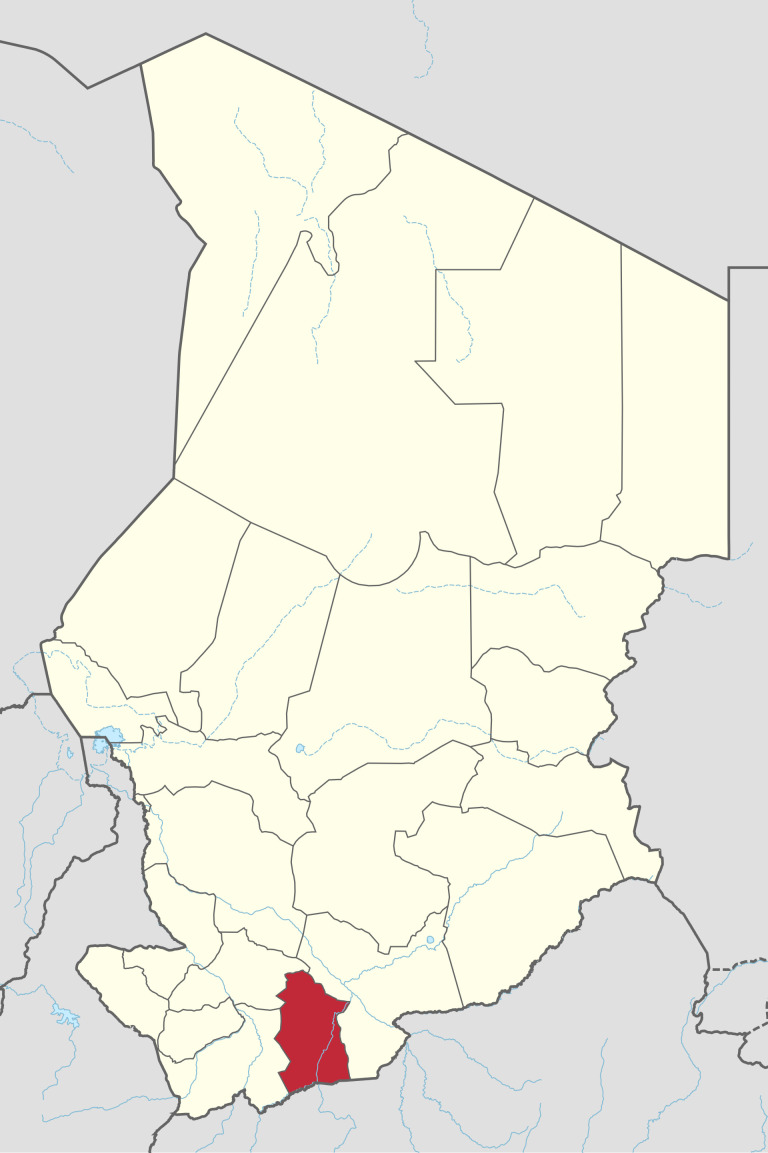
Map of Mandoul within Chad.

Previously described by Mahamat et al. (13), the Mandoul region covers about 840 km^2^ in parts of five cantons in Southern Chad (Fig. 8). It is a historical focus of g-HAT due to *T. brucei gambiense*, transmitted in the area by only one species of tsetse, *G. f. fuscipes*. The mean elevation is ~400 m and annual rainfall is between 1,000 and 1,200 mm: a wet season lasts from June to October and a dry season from November to May. Vegetation, consisting of woody savannah with gallery forest along the rivers, has been degraded in parts through agriculture. The population comprises pastoralist livestock keepers and sedentary mixed crop-livestock farmers—cultivating sorghum, sesame, and sweet potatoes.

#### Vector surveillance.

**Fig. 9.**
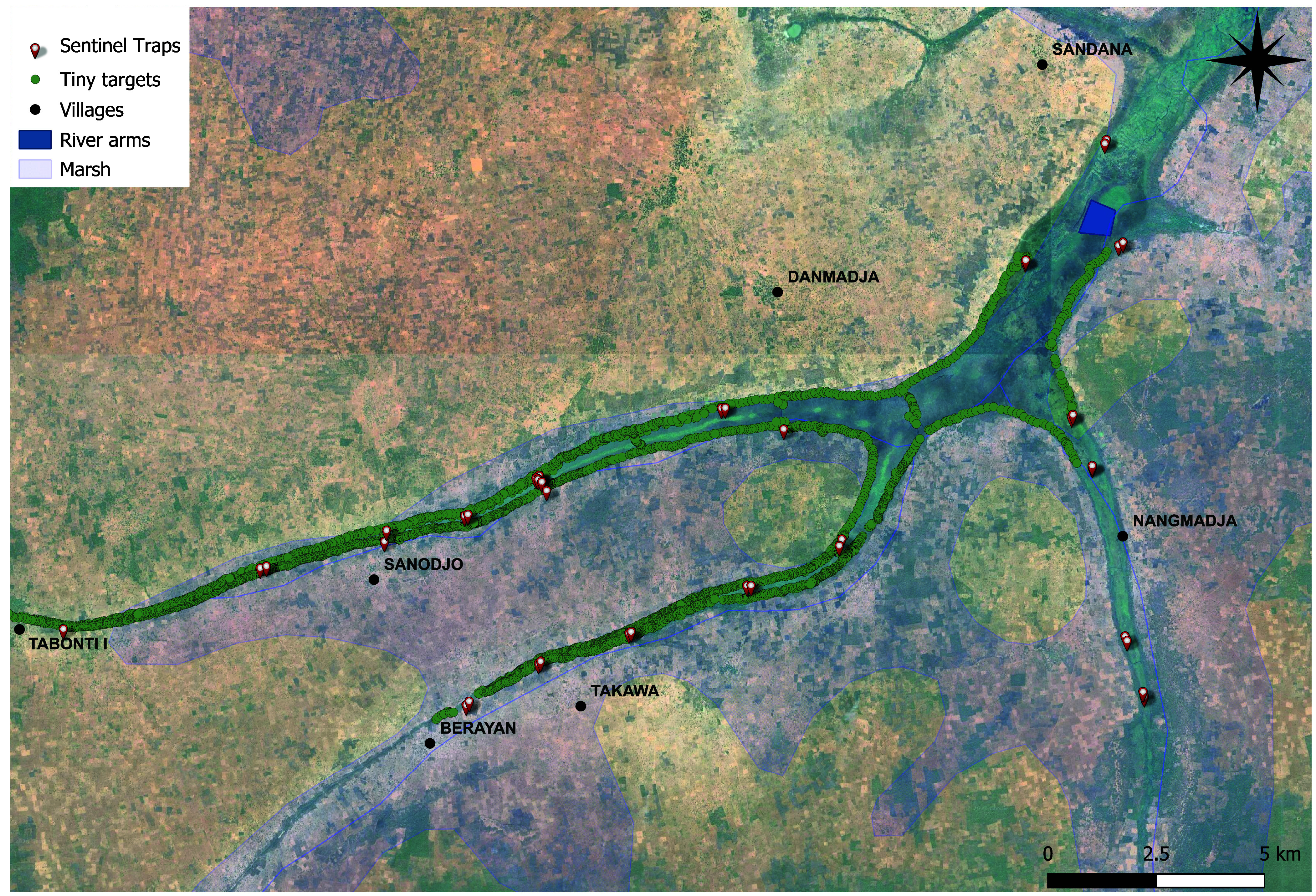
Map showing the deployment positions of the 44 biconical traps used for surveillance and the locations of the 2,713 tiny targets in Mandoul from 2014.

In November 2013, prior to target deployment, the above-cited baseline survey involved the deployment of 108 biconical traps (54) across the whole Mandoul area. The traps were left in situ for 48 h, and the numbers of *G. f. fuscipes* captured in each trap were then recorded. Thereafter, sampling traps were deployed only at 44 sentinel sites and were operated for 2 d, twice each year, from 2014 to 2024 (Fig. 9).

Tiny targets are used to kill tsetse, whereas biconical traps are used only as a monitoring system to catch live tsetse. Hence, from 2014 to 2025, tiny targets stayed active all year long (albeit being sometimes replaced by new ones) while traps were only deployed 2 d at a time, a few times a year, and then removed.

#### Vector control (Tiny Target deployment).

**Fig. 10.**
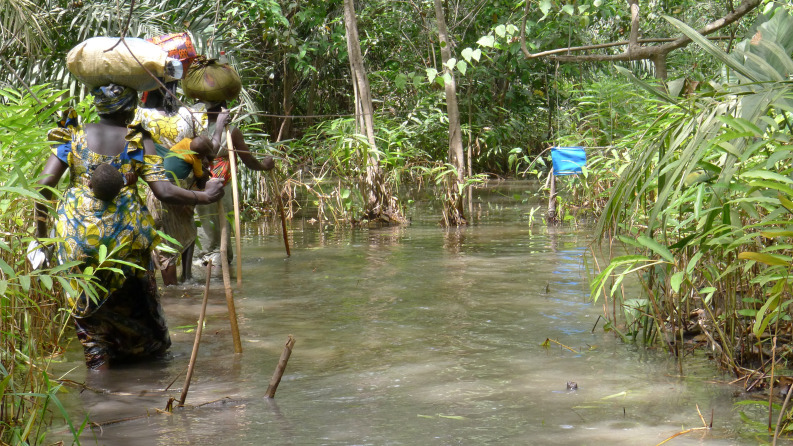
A Tiny Target—as deployed in the Mandoul focus.

Recent efforts to eliminate the Mandoul focus of g-HAT are based on the use of Tiny Targets (12) (Fig. 10), deployed after a baseline trapping survey had determined the numbers, species, and distribution of tsetse, thereby delineating the area to be controlled (13). The Tiny Targets, provided by Vestergaard (Lausanne, Switzerland), comprised 0.25 m × 0.25 m blue polyester flanked by 0.25 m × 0.25 m black polyethylene netting impregnated with deltamethrin at 300 mg/m^2^ (12). Targets were deployed along the three main arms of the Mandoul River, where tsetse were detected, and over an area up to 4 km beyond where tsetse were caught. Targets were suspended from tree branches at 10 to 20 cm above the ground, using string, or erected with wooden sticks obtained locally (Fig. 10). A total of 2,713 targets were deployed in January–February 2014 and replaced annually until 2022, whereupon 10% of them were removed in 2023, a further 40% removed in 2024, and the last targets removed in April 2025.

## Supplementary Material

Appendix 01 (PDF)

Dataset S01 (XLSX)

Dataset S02 (XLSX)

Dataset S03 (XLSX)

Dataset S04 (XLSX)

Dataset S05 (XLSX)

Dataset S06 (XLSX)

Dataset S07 (XLSX)

Dataset S08 (XLSX)

## Data Availability

All study data are included in the manuscript and/or supporting information.
