# Supplementary material for: Modeling framework to demonstrate elimination of a vector population: Tsetse elimination in Chad
Source: Proc Natl Acad Sci U S A. 2026 Jun 23;123(26):e2524729123. doi: 10.1073/pnas.2524729123 (PMC13320919; doi:10.1073/pnas.2524729123)
Supplement: Supplementary file 1 — Appendix 01 (PDF) [file pnas.2524729123.sapp.pdf]

SI Appendix

Modeling framework to demonstrate elimination of a vector population: tsetse elimination in Chad

John Hargrove[1], Mahamat Hissene Mahamat[2], Moukhtar Aldjibert[2], Wilfrid Yoni[3], Djoukzoumka Signaboubo[2], Justin Darnas[4], Ernest Salou[3,5], Inaki Tirados[6], Albert Mugenyi[7], Priscille Barreaux[8], Philippe Solano[9], Antoine Marc Gaby Barreaux[9,10,11,*]

[1]South African Centre for Epidemiological Modelling and Analysis (SACEMA), Centre for Epidemic Response and Innovation (CERI), School for Data Science and Computational Thinking, Stellenbosch University, Stellenbosch, South Africa
[2]IRED, Ndjamena, Chad
[3]CIRDES, Bobo-Dioulasso, Burkina Faso
[4]PNLTHA, Chad
[5]Université Nazi Boni de Bobo-Dioulasso (UNB), Burkina-Faso
[6]LSTM, Liverpool, United Kingdom
[7]Ministry of Agriculture Animal Industry and Fisheries, Kampala, Central, UG
[8]ICIPE, human health theme, Nairobi, Kenya
[9]INTERTRYP, Université de Montpellier, IRD, CIRAD, Montpellier, France
[10]Cirad, UMR INTERTRYP, F-34398 Montpellier, France
[11]ICIPE, Animal health theme, Nairobi, Kenya
*Corresponding author:
Antoine Marc Gaby Barreaux
Animal health Theme, ICIPE
International Centre of Insect Physiology and Ecology (icipe)
P.O. Box 30772-00100 Nairobi, Kenya

Email: antoine.barreaux@cirad.fr

This PDF file includes:

Supporting text

Figures S1 to S2

Legends for Datasets S1 to S8

SI References

Other supporting materials for this manuscript include the following:

Datasets S1 to S8

1. Annual growth of a tsetse population as a function of adult mortality and inter-larval period

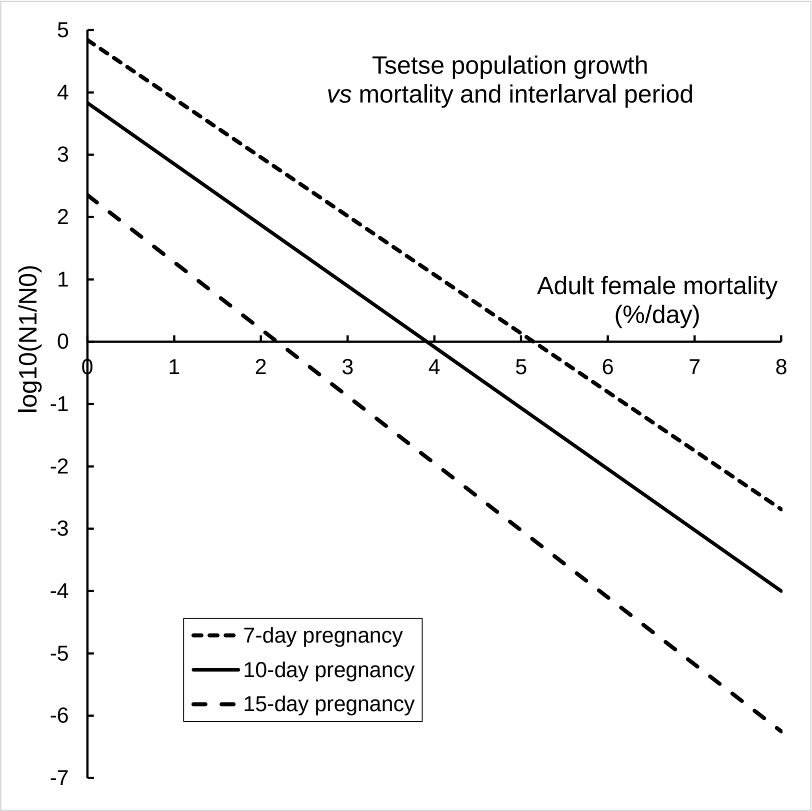

Figure S1. Annual growth of a tsetse population as a function of adult mortality and inter-larval period (pregnancy duration). Growth is expressed as the number of flies (*N*1) alive at the end of a year divided by the number present at the beginning of the year (*N*0). Redrawn from (1).

2. The six-step approach in Mandoul

Step 1) Baseline probability of capture

Although excellent baseline work, including a comprehensive baseline survey, was carried out in Mandoul, this was a control operation, not a research study – and no trials were carried out to estimate the efficacy of the traps and targets used in the control exercise. Moreover, neither life history nor population dynamics studies were carried out on the population of *G. f. fuscipes* in the Mandoul area. Accordingly, as detailed below, we rely on theory and past examples from other study sites to parametrize some of our models to obtain the probability of capturing a tsetse with the biconical traps used in Mandoul.

We first estimate the theoretical probability that a given vector control method could, in theory, successfully eliminate an isolated population of tsetse (2). We then estimate the probabilities of kill/capture after deployment of targets/traps for a series of days in Mandoul. Finally, using information from Big Chamaunga Island, Kenya, we estimate the probability *p*

that a female *G. f. fuscipes*, alive in the Mandoul at the start of a given day, is killed by a target or captured by an individual biconical trap. We assume that we are indeed dealing with an isolated population in Mandoul, since the nearest tsetse population sampled beyond the borders of the Mandoul focus is ~50km distant (3).

Hargrove (2005) (2) calculated the probability (s) of eliminating an isolated tsetse population, as a function of the variables impacting female birth and death rates. The probability is given by the solution of the equation:

$$P(elimination) = (B\gamma+M+B \pm \mathrm{sqrt}(B\gamma+M+B)^2 - 4B(M+B\gamma))/2B \qquad (1)$$

where, $\gamma = 1 - \eta\psi(b)^{\tau(b)}$, $B = \alpha\psi(c)^{\tau(c)}\psi(a)^{\tau(a)}$ and $M = 1 - \psi(c)^{\tau(c)}$

See Table 1 for the definition of all parameters and variables. The probability of elimination is the smaller of the two roots of equation (1). This gives the probability that the line emanating from a single female fly is eliminated. If generation zero consists of N flies, all subject to the same survival probabilities and reproductive rates, the whole population is eliminated with probability s^N.

Evaluation of equation (1) shows that, if the mortality of adult female tsetse in an isolated population can be maintained at a level of at least 3.5 – 4.0% per day, that population will be eliminated with probability 1.0 (Figure S2) – even if there are no reproductive losses, and regardless of density dependent effects (2). The minimum mortality required to be sustained among adult females, in order to achieve elimination, naturally decreases as pupal mortality increases. Thus, if pupal mortality is of the order of at least 1% per day, as estimated for *G. pallidipes* in Kenya (4), a sustained adult mortality of between 2.5 and 3.0% per day will ensure elimination (Figure S2).

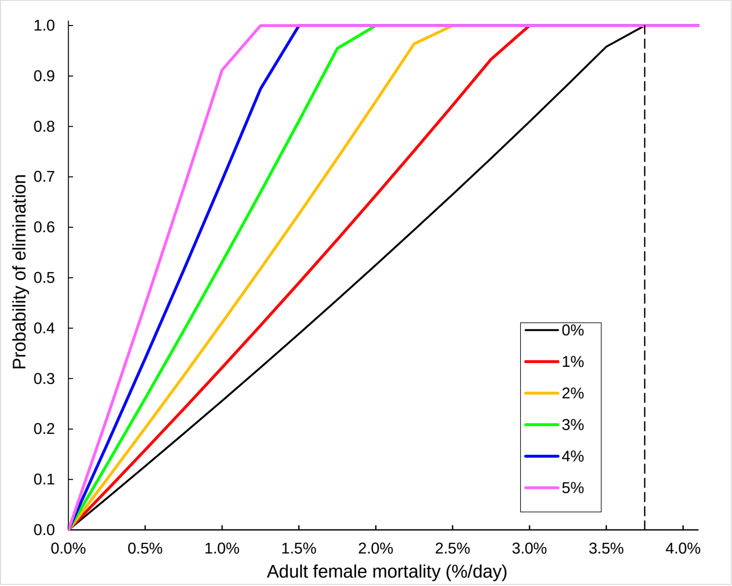

Figure S2. Probability of elimination of an isolated tsetse population as a function of the mortality of female adult females and pupae (legend from 0 to 5%). Calculated for values of u = 7 days, I = 9 days, and T = 27 days. Redrawn from (2). Details of calculations in Dataset S7.

Probabilities of kill/capture after deployment of targets/traps for a series of days in Mandoul

For the Chad case study, assume that there is a probability p that a female *G. f. fuscipes* alive in the Mandoul at the start of a given day is killed by a target during that day. Then the probability that this fly is *not* killed by a target during that day is $q = 1 – p$. In what follows we make frequent use of the assumption that the probability that a fly is *not* killed by any target on a given day is independent of the probability that it was *not* killed by any target on the previous day. By extension, this assumption means that the fly escapes being killed by a target on n consecutive days with probability $Q = q^n$ – and the probability that the fly has been killed by day n is $P = 1 – Q = 1 - q^n$. An analogous argument applies to the probabilities that a fly is captured, or evades capture, by any given trap.

Estimate probability p that a female G. f. fuscipes, alive in the Mandoul at the start of a given day, is killed by a target/captured by an individual trap – using information from Big Chamaunga Island, Kenya

The probabilities of trapping *G. f. fuscipes*, or killing them using Tiny Targets, were estimated using data from Tirados et al. (5), who monitored the decline in trap catches of *G. f. fuscipes*, following the deployment of Tiny Targets on Big Chamaunga Island, (-0.426° latitude, 34.233° longitude; surface area 0.2km^2; circumference 1.5 km), which lies in the Kenyan section of Lake Victoria. From January 2011 - December 2012, they deployed 30 Tiny Targets at 50m intervals in the shoreline habitat of the island, giving a target density of 20 targets/km. They monitored the impact of deploying targets along the island shore via monthly catches of tsetse, from four biconical traps deployed, between 200 and 300m apart, along the lakeshore, and a further single trap placed at the center of the island.

Step 2) Probability of elimination based on vector capture

We apply a probability model to the results of our surveillance efforts to reject the null hypothesis that insects are still present following a series of days in Mandoul with no tsetse being captured.

We define:

- *A* Area sampled (km^2), assumed isolated (closed to immigration and emigration).
- *N* Total insects surviving the eradication attempt, assumed randomly distributed in *A*.
- ε Trap efficiency, *i.e.*, the conditional probability that an insect is caught by a given trap, given that there is only one trap present in the 1-km^2 square containing the insect and given that the insect is active.
- *S* Number of traps present in all of *A*.
- *t* Number of days for which each trap is operated.

With these definitions Hargrove (6) showed that the probability of capturing at least one fly is approximately:

$$C(N, S, \varepsilon, t) = 1 - \exp(-StN\varepsilon/A) \approx StN\varepsilon/A \quad (2)$$

the approximation holding for populations close to elimination, when the exponent is small.

Hence the probability (C′) of null trapping, *i.e.*, of catching no tsetse at all, is

$$C'(N, S, \varepsilon, t) = \exp(-StN\varepsilon/A) \approx 1 - StN\varepsilon/A \quad (3)$$

We aim to minimize the risk (α) of falsely concluding – from a sequence of zero catches – that tsetse have been eliminated when, in fact, there are still tsetse present. Accordingly, we typically set α to some value close to zero; say, $\alpha = 0.01$ or $\alpha = 0.001$. Then, if we find that $C' \approx 1 - StN\varepsilon/A < \alpha$, we conclude that tsetse have been eliminated, with the attendant risk, α, that our conclusion is false.

Step 3) Probability of natural elimination

We estimate the probability that a very small residual population will be eliminated by chance. If only a small number of tsetse survive a control operation, there is a non-zero probability that this remnant population will be eliminated by chance – without the need for further control efforts. The probability that this will occur is calculated using Equation (1) – with the assumption that mortalities among adult and immature females can be much lower than when the population is subjected to control measures. Figure 3 (Main text) shows that even if only one inseminated female survives, and if the background adult female mortality is 2% per day there is still a 40% chance that the female will give rise to a surviving population. If there are 10 surviving inseminated females, the population will be almost certain to survive – even if adult female mortality is 2.5% per day.

Step 4) Detection of potential rebound

If there is insufficient evidence – even after a series of zero catches of tsetse – to conclude that tsetse have been eliminated, or that the remaining small tsetse population may be eliminated naturally, then there are two options.

The first, Step 2´, is to keep vector control/Tiny Targets in place and to continue sampling with traps until the sequence of zero catches is so long that we can have a high degree of confidence that elimination has been achieved. Hence, using Equation 2, one calculates the sampling duration needed before reaching a high confidence (below the risk ratio) that the vector population has been eliminated. If feasible one could calculate a combination of increased sampling efforts (more traps) and prolonged surveillance efforts. This has the advantage that the presence of Tiny Targets will ensure a low risk of a recurrence of cases of human trypanosomiasis. It has the disadvantage, however, of incurring, for an unknown/long period, the continued costs of employing the control team, and buying and deploying new Tiny Targets. Moreover, these costs are wasted if the tsetse population has, in fact, already been eliminated.

The alternative is to remove all Tiny Targets from the control area, here Mandoul, and to continue with the vector sampling effort – but to stop all control measures and aim at detecting a potential rebound in vector population, which is our actual step 4. There are two possible outcomes of step 4: (i) The tsetse population has indeed already been eliminated – in which case the ongoing sampling will fail to catch a fly, regardless of how long the sampling continues: there is then no longer any need to carry out any manner of vector or disease control. (ii) The more interesting, and problematic, possibility is that the surviving tsetse are not eliminated by chance after the Tiny Targets have been removed. If this is the case, we expect the tsetse population to grow steadily, particularly given that there is no longer any risk of the flies being killed by Tiny Targets. Then one has to determine whether vector control should be restarted.

Failure to detect a rebound in the vector population after the cessation of control efforts will support the conclusion that the vector has already been eliminated. A growth model is used to estimate the expected vector population at various times after the cessation of control efforts, assuming the survival of at least one reproductive female. If no tsetse can be captured, despite predictions of a large population from the growth model, then the vector population can be considered as eliminated.

The growth of an isolated population of tsetse is determined by the balance between the rates of larval production, and development – and by the rates of immature and adult mortality, whether natural or imposed by human intervention. Hargrove (1) estimated growth rates of tsetse populations, as functions of birth and death rates, by calculating dominant eigenvalues of appropriate Leslie matrices. Those results, summarized in Figure 4 (Main text), are valid for any tsetse population; it is only necessary to stipulate the appropriate levels of the birth and death rates. In setting development rates for tsetse in the Mandoul area, we use a default mean daily temperature of 28.7°C. (Dataset S8). In the absence of field estimates of the effects of temperature on various development rates in *G. f. fuscipes,* we use relationships measured for *G. m. morsitans* and *G. pallidipes,* which deposit their first larva at the age of about 14 days, and subsequent larvae at 8-day intervals (6–8). Pupal duration for female *G. m. morsitans,* at a constant temperature of 28.7°C in the laboratory, is *ca*.21 days (9, 10). In the field, however, temperatures in typical larviposition sites are about 2°C cooler on average than ambient (11, 12). Accordingly, we assume a temperature of 26.7°C during pupal

development, giving an expected pupal duration of *ca*.23 days. We estimate possible growth rates for tsetse populations in the Mandoul area, before and after the use of Tiny Targets, for a wide range of adult female mortalities (Figure 4, Main text). Having predicted the growth of the tsetse population, one can then use Equations (2) and (3) of step 2 to determine when we will have a 90%, 99% or 99.9% confidence that our zero catches, and failure to detect a rebound, actually means elimination.

Step 5) Vector elimination and reinvasion risk

Finally, we evaluate whether the vector population has been successfully eliminated with a specified level of risk, while also accounting for the potential threat of reinvasion. The above methodologies, for estimating the probability of elimination of a tsetse population, apply to situations where the tsetse population is isolated. If, however, we conclude that tsetse have been eliminated from the Mandoul, we still need to estimate the probability that the area could be repopulated by tsetse invading from distant populations. The nearest tsetse population, beyond the borders of the Mandoul focus is ~50km distant (3).

Dispersal in tsetse is generally modelled as a random walk (13–15) or, equivalently, as a diffusion process (16). This last paper was published only as a hard copy and is not generally available. Accordingly, it is included here as Supplementary File S10 and is used to estimate the probability that a tsetse fly, present at time 0 at a random point in a given area, will be found in some distant neighborhood at time *t* later, given that it is still alive. In making these calculations for *G. f. fuscipes*, we use – as a first approximation – Rogers' (14) estimate that *G. f. fuscipes* moves an average of 137 m (150 yds) per day and assume that this rate of movement does not vary significantly with age.

The closest population of tsetse, *G. f. fuscipes*, beyond the borders of the Mandoul focus is in the neighborhood of Timbéri, some 50 km distant. We estimate the probability that a tsetse fly could survive long enough to move between the two populations. We use the results of Hargrove & Lange (16) in modeling tsetse dispersal as diffusion in the plane, starting at the origin when time $t = 0$, with coefficient of diffusion σ^2. The fly's position (x, y, t) in time and space is then defined by a normally distributed random variable with density function

$$f(x,y,t) = (1/(2\pi g)) \exp(-(x^2 + y^2)/2g) \qquad (4)$$

where $$g = g(t) = \int_0^t \sigma^2(s)\, ds$$

As a first approximation we assume σ^2 is independent of the fly's age and position in the plane, so that $g = kt$, where k is a constant coefficient of diffusion. Consider a fly starting its dispersal at a point chosen uniformly from the interval [a, b]. Then at some time t it will be in the interval [c, d] with probability:

$$P\,(c, d, t) = 1/(b-a)\,[\Phi\,((d-z)/\sqrt{kt})\,(z-d) - \Phi\,((c-z)/\sqrt{g})(z-c)$$

$$+ \sqrt{kt/2\pi}\,(\exp(-(c-z)^2/2kt) - \exp(-(d-z)^2/2kt))\,]_a^b \qquad (5)$$

If the intervals [a, b] and [c, d] are widely separated, relative to the rate of diffusion, such that $(c-a)/(kt)^{0.5} >> 0$, then Equation (5) can be much simplified because:

$$\Phi((c-a)/(kt)^{0.5} \approx 1$$

$$\exp(-(c-a)^2/2kt) \approx 0$$

Step 6) Sensitivity analysis

We investigate how our conclusions would be impacted by variations in the assumed values of the various development rates and survival probabilities (Table 1 main text). Central to this analysis is the following equation (17) relating growth rates to the variables influencing development rates and survival probabilities at various life stages:

$$\beta e^{[(\mu(a) - r)\tau(a) + (\mu(b) - r)\tau(b) + (\mu(c) - r)\tau(c)]} = 1 - e^{[(\mu(c) - r)\tau(c)]} \qquad (6)$$

where r is the growth rate, β is the fecundity, counting female larvae only; $\tau(a)$, $\tau(b)$ and $\tau(c)$ are the durations of the pupal period, the nulliparous stage and the inter-larval period, respectively (time between the deposition of each larva as tsetse don't lay eggs but deposit a live larva, one at a time); $\mu(a)$, $\mu(b)$ and $\mu(c)$ are the survivorships during these periods. See Table 1 for the formal definitions, and allowed ranges, of all variables and parameters. All parameters, other than β and $\tau(b)$, are temperature dependent. Mandoul is close to the Equator and shows limited temperature variation across the year (Supplementary File S10). The mean daily temperature, over all months of the year, is 28.7°C (standard deviation 2.1°C). The lowest monthly mean (25.2°C in January) is 3.5°C lower than the mean, and the highest (33.5°C in April) 4.6°C higher. We estimate below the effects of varying mean temperatures between 24 and 32°C. The upper bound is more than adequate, given that constant temperatures of 30°C can cause sterility in *G. f. fuscipes* (18) and *G. m. morsitans* pupae kept in the laboratory at any constant temperature >32°C all die before emerging (11). The inclusion of the high upper bound has the advantage that it makes a liberal allowance for possible increases in temperature that may have occurred in the past few decades, reflecting global increases in temperature.

β, the fecundity, is defined as the number of female pupae produced by an adult female at each pregnancy: this will be 0.5, less any losses that occur during uterine development. Such losses have been estimated by various workers (6, 19–21) though none used *G. f. fuscipes*. Reference (6) provides the most complete study, being based on the ovarian dissection of >170,000 adult female *G. pallidipes* and *G. m. morsitans* captured in the field in Zimbabwe. Abortion rates of <1% per pregnancy were estimated in 11 of the 12 months of the year; higher rates observed in other studies may have been due, in part, to trauma experienced by the pregnant female during capture. Nonetheless, we allow the possibility that abortion rates could vary between 0 and 5% per pregnancy. so that β varied between 0.5 and 0.475.

There are no published data for *G. f. fuscipes* on development rates as measured in the field: we thus used data for other species, often studied only in the laboratory. These studies suggest that there is little variation between species in developmental rates at the same temperature (22). Accordingly, we use rates estimated in Zimbabwe for *G. m. morsitans* and *G. pallidipes* (10, 23). These results predict pupal durations, $\tau(a)$, of 19 and 29 days, and pregnancy durations, $\tau(c)$, of 8 and 10 days, for mean temperatures of 32 and 24°C, respectively.

Rogers & Randolph (1984) (24) used *G. f. fuscipes* to estimate tsetse pupal mortality, $\mu(a)$, in the field, in Uganda, finding density dependent losses increasing from 1% per day at low pupal density, to >5% per day when pupal density exceeded $32/m^2$. There are no field data on the effect of temperature on pupal mortality. For *G. m. morsitans* pupae maintained in the laboratory mortalities were <1% day, independent of temperatures between 20 and 30°C (11). Given these results, and the fact that densities of *G. f. fuscipes* were clearly very low even before Tiny Targets were deployed, we investigate the effects of varying pupal loss rates,

μ(*a*), between 0 and 2% per day. The variation with temperature of τ(*b*), the time between an adult female emerging and her first ovulation, is sufficiently small that the parameter is often set at a constant 8 days (17), though there is some variability about this value (25). Accordingly, we allow τ(*b*) to vary between 7 and 9 days. Mark-recapture experiment, using an island population of *G. m. morsitans*, demonstrated that mortality (μ(*b*)) among young females was significantly higher than for older flies μ(*c*) (26, 27). No other study has ever demonstrated age-dependent mortality in the field, but the results is consistent with laboratory data. Accordingly, we set μ(*b*) = *Y*×μ(*c*) and allow *Y* to take values between 1 and 3.

3. The different data sets used to produce figures and calculations for the six steps (separate files):

- Dataset S1_figure 1_ Probability of capture for Chamaunga and Mandoul.xlsx
- Dataset S2_figure 2_ Probability of zero catches.xlsx
- Dataset S3_figure 3_ Probability eliminated by chance.xlsx
- Dataset S4_figure 4_ Probability of catching single fly in 68 km.xlsx
- Dataset S5_figure 5_ Probability of Zero catch in Mandoul increased effort.xlsx
- Dataset S6_figure 6_ Mandoul tsetse growth rates.xlsx
- Dataset S7_figure S2_ Probability of Elimination v Adult and Pupal Mortalities and Pioneer population.xlsx
- Dataset S8_For sensitivity analysis_Mandoul temperature data 2000 to 2025.xlsx

4. SI References

1. J. W. Hargrove, Tsetse: the limits to population growth. *Med. Vet. Entomol.* **2**, 203–217 (1988).

2. J. W. Hargrove, Extinction probabilities and times to extinction for populations of tsetse flies Glossina spp. (Diptera: Glossinidae) subjected to various control measures. *Bull. Entomol. Res.* **95**, 13–21 (2005).

3. S. Ravel, *et al.*, Population genetics of Glossina fuscipes fuscipes from southern Chad. *Peer Community J.* **3**, e31–e31 (2023).

4. D. J. Rogers, S. E. Randolph, Estimation of rates of predation on tsetse. *Med. Vet. Entomol.* **4**, 195–204 (1990).

5. I. Tirados, *et al.*, Tsetse control and Gambian sleeping sickness; implications for control strategy. *PLoS Negl. Trop. Dis.* (2015). https://doi.org/10.1371/journal.pntd.0003822.

6. J. W. Hargrove, Tsetse eradication: sufficiency, necessity and desirability. *DFID Anim. Health Programme* 133 + ix pp. (2003).

7. J. W. Hargrove, Reproductive rates of tsetse flies in the field in Zimbabwe. *Physiol. Entomol.* **19**, 307–318 (1994).

8. J. W. Hargrove, Towards a general rule for estimating the stage of pregnancy in field-caught tsetse flies. *Physiol. Entomol.* **20**, 213–223 (1995).

9. R. J. Phelps, P. M. Burrows, Puparial duration in Glossina morsitans orientalis under conditions of constant temperature. *Entomol. Exp. Appl.* **12**, 33–43 (1969).

10. J. W. Hargrove, G. A. Vale, Models for the rates of pupal development, fat consumption and mortality in tsetse (Glossina spp). *Bull. Entomol. Res.* 1–13 (2019). https://doi.org/10.1017/S0007485319000233.

11. R. J. Phelps, P. M. Burrows, Lethal temperatures for puparia of Glossina morsitans orientalis. *Entomol. Exp. Appl.* **12**, 23–32 (1969).

12. P. J. Jackson, R. J. Phelps, Temperature regimes in pupation sites of Glossina morsitans orientalis Vanderplank (Diptera). *Rhod. Zamb. Malawi J. Agric. Res.* **5**, 249–260 (1967).

13. E. Bursell, "Dispersal and concentration of Glossina" in *The African Trypanosomiases*, H. W. Mulligan, Ed. (George Allen and Unwin, 1970), pp. 382–394.

14. D. Rogers, Study of a natural population of Glossina fuscipes fuscipes Newstead and a model of fly movement. *J. Anim. Ecol.* **46**, 309–309 (1977).

15. J. W. Hargrove, Tsetse dispersal reconsidered. *J. Anim. Ecol.* **50**, 351–351 (1981).

16. J. W. Hargrove, K. Lange, Tsetse dispersal viewed as a diffusion process. *Trans. Zimb. Sci. Assoc.* **64**, 1–8 (1989).

17. B. G. Williams, R. D. Dransfield, R. Brightwell, Tsetse fly (Diptera: Glossinidae) population dynamics and the estimation of mortality rates from life-table data. *Bull. Entomol. Res.* **80**, 479–485 (1990).

18. H. Mellanby, Experimental work on reproduction in the tsetse fly, Glossina palpalis. *Parasitology* **29**, 131–141 (1937).

19. L. C. Madubunyi, Relative frequency of reproductive abnormalities in a natural population of Glossina morsitans morsitans Westwood (Diptera: Glossinidae) in Zambia. *Bull. Entomol. Res.* **68**, 437–442 (1978).

20. D. A. Turner, W. F. Snow, Reproductive abnormality and loss in natural populations of Glossina pallidipes Austen (Diptera: Glossinidae) in Kenya. *Bull. Entomol. Res.* **74**, 299–309 (1984).

21. J. W. Hargrove, Nutritional levels of female tsetse Glossina pallidipes from artificial refuges. *Med. Vet. Entomol.* **13**, 150–164 (1999).

22. S. G. A. Leak, *Tsetse biology and ecology: their role in the epidemiology and control of trypanosomosis.* (CAB International, in association with the International Livestock Research Institute, Nairobi, Kenya, 1998).

23. J. Hargrove, "Tsetse population dynamics" in *The Trypanosomiases*, I. Maudlin, P. Holmes, M. Miles, Eds. (CABI Publishing, 2004), pp. 113–135.

24. D. J. Rogers, S. E. Randolph, A review of density-dependent processes in tsetse populations. *Int. J. Trop. Insect Sci.* **5**, 397–402 (1984).

25. J. W. Hargrove, Age-specific changes in sperm levels among female tsetse (Glossina spp.) with a model for the time course of insemination. *Physiol. Entomol.* **37**, 278–290 (2012).

26. J. W. Hargrove, Age-dependent changes in the probabilities of survival and capture of the tsetse, Glossina morsitans morsitans Westwood. *Int. J. Trop. Insect Sci.* **11**, 323–330 (1990).

27. J. W. Hargrove, R. Ouifki, Ameh, J. E., A general model for mortality in adult tsetse (Glossina spp.). *Med. Vet. Entomol.* **25**, 385–394 (2011).
